# Core Binding Factor Leukemia: Chromatin Remodeling Moves Towards Oncogenic Transcription

**DOI:** 10.3390/cancers11121973

**Published:** 2019-12-07

**Authors:** Alessandro Beghini

**Affiliations:** Department of Health Sciences, University of Milan, 20142 Milan, Italy; alessandro.beghini@unimi.it; Tel.: +39-503-223-230

**Keywords:** core binding factor leukemia, AML, RUNX1, RUNX1T1, CBFB, MYH11, miRNA, chromatin remodeling

## Abstract

Acute myeloid leukemia (AML), the most common acute leukemia in adults, is a heterogeneous malignant clonal disorder arising from multipotent hematopoietic progenitor cells characterized by genetic and concerted epigenetic aberrations. Core binding factor-Leukemia (CBFL) is characterized by the recurrent reciprocal translocations t(8;21)(q22;q22) or inv(16)(p13;q22) that, expressing the distinctive *RUNX1-RUNX1T1* (also known as Acute myeloid leukemia1-eight twenty-one, AML1-ETO or RUNX1/ETO) or *CBFB-MYH11* (also known as CBFβ-SMMHC) translocation product respectively, disrupt the essential hematopoietic function of the CBF. In the past decade, remarkable progress has been achieved in understanding the structure, three-dimensional (3D) chromosomal topology, and disease-inducing genetic and epigenetic abnormalities of the fusion proteins that arise from disruption of the CBF subunit alpha and beta genes. Although CBFLs have a relatively good prognosis compared to other leukemia subtypes, 40–50% of patients still relapse, requiring intensive chemotherapy and allogenic hematopoietic cell transplantation (alloHCT). To provide a rationale for the CBFL-associated altered hematopoietic development, in this review, we summarize the current understanding on the various molecular mechanisms, including dysregulation of Wnt/β-catenin signaling as an early event that triggers the translocations, playing a pivotal role in the pathophysiology of CBFL. Translation of these findings into the clinical setting is just beginning by improvement in risk stratification, MRD assessment, and development of targeted therapies.

## 1. Introduction

The year 2016 coincided with the 25th anniversary of the first cloning of mammalian Runt (Runt domain)-related transcription factor 1 (*RUNX1*) gene, associated with hematologic disorders [1]. *RUNX1* (AML1) is a master transcriptional regulator of adult hematopoiesis also involved in the establishment, maintenance, and functional integrity of hematopoietic stem cells (HSCs) in embryonic and adult blood compartments [2,3,4]. AML1 post-translational modifications help create scaffolds that interact and bind with multiple members recruited to the core binding factor (CBF), promoting or repressing transcription. At about the same time, the gene encoding *CBFB* (CBFβ) was identified as disrupted by the inv(16) in acute myeloid leukemia [5]. Normally, AML1 and CBFβ form a DNA-binding heterodimer required for binding to the consensus sequence, where it recruits lineage-specifying transcription factors to regulate hematopoietic differentiation. As the Runt-related transcription factor (RUNX) gene family plays important roles in tissue-specific gene expression, it is frequently involved in the malignant transformation of the hematopoietic system. Acute leukemias characterized by the presence of t(8;21) or inv(16) are defined core-binding factor Leukemias (CBFLs), since they both alter the CBF transcription factor complex [6]. Approximately 30% and 13–15% of newly diagnosed pediatric and adult AML patients, respectively, are diagnosed as CBFLs [7]. Although the CBFLs are categorized into a favorable-risk group as compared with other subtypes of AML, approximately 30–40% of the patients still relapse and may require allogeneic hematopoietic cell transplantation (HCT) [8,9]. *RUNX1–RUNX1T1* and *CBFB–MYH11* translocations may represent acquired initiating events occurring in hematopoietic progenitors. However, little is known about the molecular mechanisms that drive the generation of the t(8;21) or inv(16), after which leukemia clonally evolves through accumulation of secondary mutations. The hypothesis that Wnt signaling promotes genomic proximity between *RUNX1* and *RUNX1T1* has been recently examined by experiments establishing that Wnt/β-catenin signaling supports *RUNX1* and *RUNX1T1* expression in hematopoietic precursors and provides spatial information, indicating that transcription of these genes is likely occurring into RNA-polymerase-II nuclear factories (RNAPII-Ser5) [10]. These results suggest a Wnt-mediated model in which an upstream molecular mechanism is capable of favoring and guiding the translocation event [11]. The incremental improvements in understanding the genetic and molecular basis of CBFLs and their association with distinct clinical and biological features provide insights into previously unappreciated cooperating pathways [12,13]. At diagnosis, the disease consists of heterogeneous clusters of cells widely differing from one another in terms of additional genetic lesions, besides sharing the specific chromosomal translocations. Cytogenetic abnormalities that alter the function of the CBF are often associated with specific receptor tyrosine kinase (RTK) mutations, suggesting that additional genetic abnormalities have an essential role in CBFL pathogenesis [14,15]. Despite a common molecular alteration involving a component of the CBF transcription complex, AMLs expressing *RUNX1-RUNX1T1* or *CBFB-MYH11* alterations display a remarkably different genome-wide spectrum of cooperating mutations [14]. Recent studies clearly indicate that AMLs with t(8;21)(q22;q22) and AMLs with inv(16)(p13q22) show different biological and clinical characteristics, supporting the notion that they represent two distinct diseases [7,16]. A series of concomitant evidence in the CBFL proved the existence of a preleukemic phase confirmed by a prolonged latency observed in experimental models between the occurrence of *RUNX1*-*RUNX1T1* CBF translocation and the development of overt leukemia [17,18], the persistence of CBFL translocations in normal HSC detected from patients in remission [19,20,21], and the maintenance of *RUNX1*-*RUNX1T1* at diagnosis and at relapses. *NRAS* (neuroblastoma RAS viral oncogene) is the most frequently mutated gene in CBFL, and over 60% of the cases harbor activating mutations in *NRAS*, *KIT* (v-kit Hardy-Zuckerman 4 feline sarcoma viral oncogene homolog), *FLT3* (FMS-like tyrosine kinase 3), *KRAS* (Kirsten rat sarcoma 2 viral oncogene homolog), *PTPN11* (protein tyrosine phosphatase non receptor type 11), and/or loss-of-function mutations in *NF1* (neurofibromin1) [9,14,15]. Integrated mutational analysis of the genetic and epigenetic changes that are relevant to the pathogenesis of CBFL would be required for a better risk stratification of patients who would benefit from dose-intensified induction chemotherapy or novel targeted therapies. AML1-ETO (eight twenty-one) (RUNX1-RUNX1T1) is the chimeric protein formed as a consequence of the t(8;21) chromosomal rearrangement, which is among the most recurrent cytogenetic rearrangements in de novo AML. The molecular mechanisms through which AML1-ETO fusion protein exerts multiple effects are not fully elucidated, yet all have focused on its strong repressor function. Moreover, several studies documented the multifunctionality of AML1-ETO fusion protein, including impaired differentiation, apoptosis inhibition, and signal activation for cell proliferation. This model might be oversimplified; however, there is convincing evidence supporting the hypothesis that leukemias are induced by cooperation between alterations in protein-coding genes and microRNAs (miRNAs), an entire novel epigenetic targets linked to leukemia development [22]. The consequences of altered expression and epigenetic status of miRNAs in CBF leukemias have been reported by us and other groups, unveiling that microRNAs are extensively integrated into the molecular networks that control leukemic development and progression [23,24,25,26,27,28]. Therefore, in this review, we summarize a synopsis of recent studies on comprehensive molecular profiles in CBF leukemias, providing a rationale for translation of the accumulating molecular evidence into clinical trials for better therapies to CBF leukemia patients.

## 2. Core Binding Factor Complex: A Critical Role in Hematopoietic Stem Cell Fate

**Figure 1:**
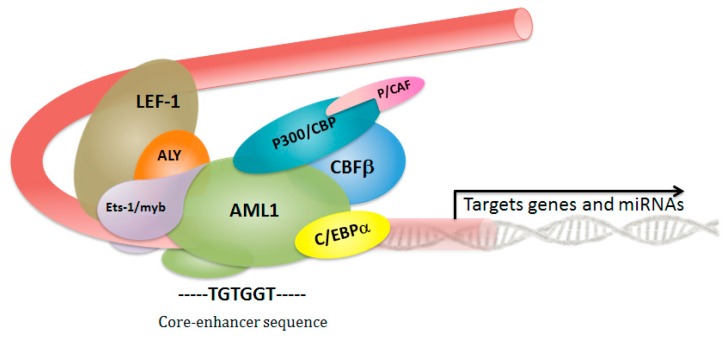
Schematic representation of the core binding factor transcriptional activation complex. DNA binding and heterodimerization with core binding factor-β (CBFβ) are mediated by the Runt domain in Acute myeloid leukemia1 (AML1 or RUNX1). The interaction with CBFβ leads to the phosphorylation of AML1, which in turn induces p300 phosphorylation, and this is mediated by homeodomain interacting kinase 2 (HIPK2) in AML1. CBP/p300 (CREB binding protein CBP and EP300); C/EBPα (CCAAT/enhancer binding protein alpha); P/CAF (P300/CBP-associated factor); AML1 (acute myeloid leukemia 1 protein); CBFβ (core binding factor subunit beta); LEF1 (lymphoid enhancer-binding factor 1); ALY (Aly/REF export factor); ETS-1 (v-ets erythroblastosis virus E26 oncogene homolog 11).

The CBF is a transcription factor complex, which consists of a distinct DNA-binding CBFα subunit (*RUNX1*, *2*, or *3*), and its non-DNA-binding heterodimerization partner CBFβ subunit (encoded by the *CBFB* gene). AML1 is a master regulatory protein expressed throughout all hematopoietic lineages. The *RUNX1* and *CBFB* genes are required for hematopoietic stem cells’ (HSCs) emergence and formation during definitive HSC development through to their terminal differentiation, and are key regulators of hematopoiesis at several steps [29,30]. The loss of definitive hematopoiesis observed in *Runx1*^−/−^ or *Cbfb*^−/−^ knockout mice and an expanded HSC compartment in conditional *Runx1*-deficient mice highlight their complex interplay in orchestrating the accurate maintenance of hematopoietic stem cell differentiation [29,30,31,32,33,34,35]. The heterodimerization with CBFβ leads to the phosphorylation of RUNX1, which in turn induces p300 (encoded by *EP300*) phosphorylation by homeodomain interacting kinase 2 (HIPK2) in AML1 [36]. By binding to the core-enhancer sequence, AML1/CBFβ complex functions as an organizing element recruiting other DNA-binding proteins, transcription factors, and co-regulators able to activate-or in some cases, repress-the target gene’s transcription. Heterodimerization with core-binding factor-β (CBFβ) confers enhanced DNA binding ability, mediated by the Runt domain in AML1. The presence of CBFβ subunit increases the affinity for DNA and, consistent with predictions, shows a significant enhancement (>40-fold enhancement) of Runt domain DNA binding of full-length AML1 (Figure 1) [37]. *RUNX1* and *CBFB* are frequent targets of gene rearrangements through chromosomal translocations and mutations that are associated with human leukemias. *RUNX1* is involved in t(8;21)(q22;q22) and t(12;21)(p13;q22) in acute myeloid and lymphocytic leukemias, and *CBFB* is rearranged in acute myeloid leukemias by inv(16)(p13;q22), t(16;16), and del(16)(q22). These cytogenetic alterations lead to the expression of fusion proteins that disrupt the heterodimeric CBF complex signaling with a dominant prevalence.

## 3. Leukemia Triggered by *RUNX1–RUNX1T1*

**Figure 2:**
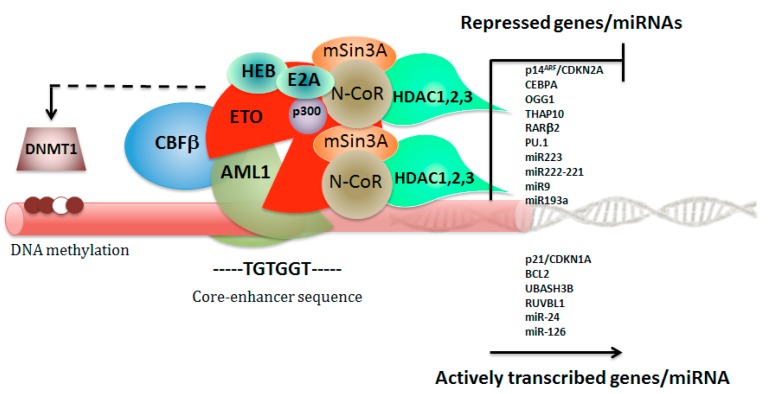
Schematic representation of the AML1–ETO (eight twenty one) repressor complex assembly. Transcriptional repression complex AML1/AML1-ETO recruits corepressors, including NCOR (nuclear receptor corepressor 1), HDACs (histone deacetylases), mSin3A (SIN3 transcription regulator family member A), and also interacts with DNMT1 (DNA methyltransferase 1) to promote DNA methylation and to repress target genes and miRNAs expression.

**Figure 3:**
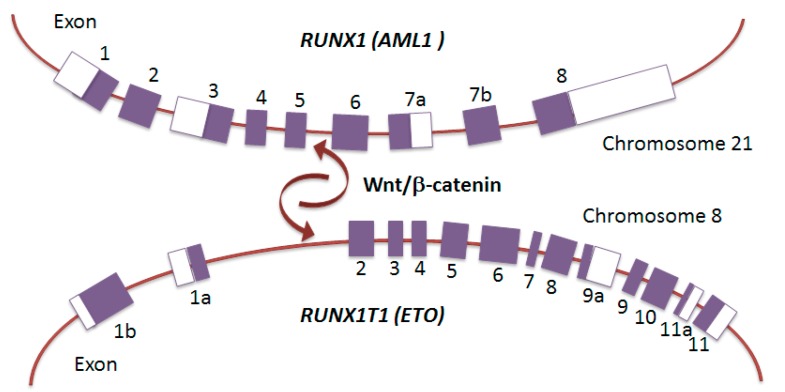
Wnt/β-catenin promotes spatial proximity and translocation of *RUNX1* and *RUNX1T1*. Genomic structure of *RUNX1* on chromosome 21 and *RUNX1T1* on chromosome 8. Wnt/β-catenin was shown to induce spatial proximity and translocation of *RUNX1* and *RUNX1T1*, which led to the generation of the *RUNX1–RUNX1T1* fusion gene. Exons are depicted as boxes.

AML1-ETO in frame fusion protein joins the N-terminal 177 amino acids of AML1 (encoded by *RUNX1*) to nearly all of ETO (encoded by *RUNX1T1*), and functions as a dominant repressor for the majority of RUNX1-responsive hematopoietic genes and microRNA (miRNA) non-coding genes. Consequently, in *RUNX1-RUNX1T1*, the DNA-binding Runt domain of RUNX1 is joined to RUNX1T1, creating a fusion transcript lacking the RUNX1 transcription activation domain. The structural insights show four essential interactions for AML1-ETO activity: DNA and CBFβ binding by the Runt domain, oligomerization through the HHR domain, and E-protein binding by the HHR domain [38,39,40,41,42]. The presence of chromosomal rearrangements, such as t(8;21) or inv(16), is associated with a unique DNA methylation patterning that predicts distinct patient outcomes, suggesting for the CBF fusion proteins also a role as epigenotype modifier [43]. The underlying mechanisms involve the AML1-ETO capacity to recruit DNA methyltransferases (DNMTs) to target tumor-suppressor genes by concerted action with certain transcriptional repressors (Figure 2) [44,45,46,47]. AML1-ETO cooperates with HIF1α to transactivate *DNMT3a* gene and shapes a positive regulatory circuit that contributes to DNA methylation signature, specifically for the non-AML1–ETO targets, leading to a DNA hypermethylation profile in AML [48]. Moreover, Ptasinska and colleagues observed that depletion of AML1-ETO and subsequent cell differentiation involves not only loss of repression, but is also associated with a redistribution of RUNX1-binding activity throughout the genome that restores, on a global scale, the epigenetic alterations mediated by the fusion protein [49]. This effect, therefore, is obtained not only through loss of repression, but involves an increased recruitment of transcription factors to additional sites, depending on AML1 interactions with other transcriptional activators, such as C/EBPα and PU.1, whose activity is altered by AML1-ETO [50,51]. ETO is a member of E-box family of transcription factors, and contains four nervy homology regions (NHR 1–4) that interact with several nuclear repressors (N-CoR, SMRT, mSin3A), including the histone deacetylases (HDACs 1–3), primarily through the NHR2 and NHR4 domains [44,52,53]. AML1-ETO also interacts with the DNA methyltransferase DNMT1 to promote DNA methylation and mediates transcriptional repression (Figure 2) [45]. However, increasing evidences from mammalian cell systems and mouse genetic models suggest that the relationship between AML1-ETO and native AML1 may be more complex, indicating that AML1-ETO depends on some functions of native AML1 to exert its proleukemogenic properties [6,54,55]. Genome-wide ChIP-Seq and RNA-Seq data recently revealed that AML1 is a member of the transcription factor complex containing AML1-ETO, and that relative binding signals on chromatin determine which genes are repressed or activated by AML1/AML1-ETO complex [56]. Thus, these new important findings indicate that the malignant cell phenotype of t(8;21) leukemia is sustained by a delicate balance between AML1-ETO and native AML1. Translocation-prone genes are preferentially recruited into transcription factories with active RNA polymerase II (RNAPII-Ser5) and need to be positioned in close spatial proximity relative to each other prior to translocation. Mechanisms that drive the generation of the *RUNX1-RUNX1T1* translocation have been poorly understood, but a recent report established that the Wnt/β-catenin signaling enhances transcription and genomic proximity of *RUNX1* and *RUNX1T1* genes, which seems to promote the generation of the *RUNX1-RUNX1T1* fusion gene [11]. These observations describe the enhanced *RUNX1T1* and *RUNX1* expression in hematopoietic precursors by Wnt/β-catenin signaling and suggest a nuclear topography of transcription likely occurring into specialized nuclear factories, thereby increasing the potential for a chromosomal translocation event (Figure 3).

## 4. Leukemia Triggered by *CBFβ-MYH11*

**Figure 4:**
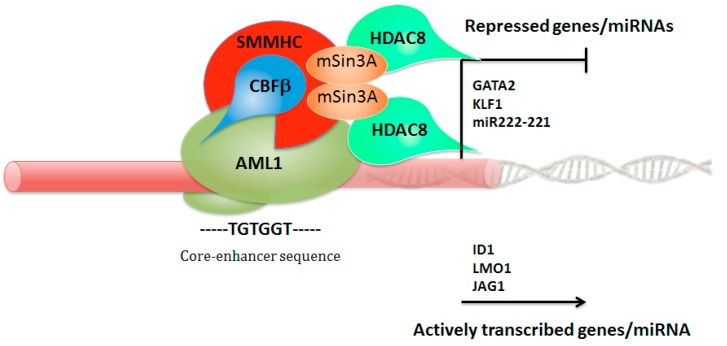
Schematic representation of transcriptional repression mediated by CBFβ-SMMHC. For transcriptional repression, CBFβ-SMMHC retains the RUNX-binding domain in CBFβ and contains an additional RUNX-binding domain in the SMMHC. Fusion protein has the ability to interact specifically with mSin3A and HDAC8 (histone deacetylase 8) through an unexpected repression domain within the SMMHC portion. GATA2 (GATA binding protein 2); KLF1 (Kruppel-like factor 1); ID1 (inhibitor of DNA binding 1); LMO1 (LIM domain only 1); JAG1 (jagged canonical notch ligand 1).

The CBFβ-SMMHC (smooth muscle myosin heavy chain, encoded by *MYH11*) fusion protein exhibits a higher binding affinity for AML1 than wild-type CBFβ. In addition to the RUNX-binding domain in CBFβ, it contains an additional RUNX-binding domain in the SMMHC portion of the fusion protein. The CBFβ-SMMHC interacts specifically with transcriptional inhibitors and HDACs, such as mSin3A and HDAC8, through an unexpected domain within the SMMHC region, thereby repressing AML1-mediated gene regulation. [57]. Therefore, CBFβ–SMMHC fusion protein acts as a transcriptional repressor, and might do so by sequestering AML1 on actin filaments in the cytoplasm. However, the majority of CBFβ–SMMHC target genes are actively transcribed, including genes such as *ID1* (inhibitor of DNA binding 1), *LMO1* (LIM domain only 1, rhombotin 1), and *JAG1* (Jagged 1) involved in hematopoietic stem cell self-renewal, and repressed upon fusion protein knockdown (Figure 4). An intrinsic inability of CBFβ-SMMHC to provide CBFβ function in vivo has also emerged, based on its failure to complete hematopoietic recovery by *Cbfb*^–/–^ mouse embryonic stem (ES) cells impaired in their capacity to generate definitive erythroid and myeloid elements [58]. Otherwise, the CBFβ–SMMHC fusion protein is predominantly recruited to promoters engaged by AML1, where it interacts with TAL1 (T-cell acute leukemia 1), FLI1 (Friend leukemia virus integration 1), and TBP-associated factors (TAFs), in synergy with a variety of hematopoietic transcription factors (ERG, GATA2, and PU.1/SPI1), as well as epigenetic coregulators, including EP300 (E1A binding protein p300) and HDAC1 (histone deacetylase 1). Recent results suggest that HDAC1 is an important component of the AML1/CBFβ-SMMHC fusion complex, which functions to activate transcription of specific target genes. The authors found that in vivo treatment with the HDAC1 inhibitor induced differentiation and apoptosis of leukemia cells, indicating HDAC1 as a potential therapeutic target [59]. These findings suggest an important role for CBFβ-SMMHC in regulating the expression of genes essential for emergence of the hematopoietic stem cell [60]. Therefore, AML1 activity is required for *CBFB-MYH11*-induced leukemogenesis [61], also through the activity of the chromodomain helicase DNA-binding protein-7 (*CHD7*), which is an ATP-dependent chromatin remodeling factor interacting with AML1/CBFβ–SMMHC complex and altering the expression of its target genes. *Chd7* deficiency in *Chd7^f/f^Mx1-CreCbfb**^+^**^/56M^* mice, which expresses the *Cbfb-MYH11* fusion gene, delayed *Cbfb–MYH11*-induced leukemia in both primary and transplanted mice [62]. Normally, the interacting interface between AML1 and CBFβ, which allows the heterodimerization [63], is retained in CBFβ-SMMHC. Moreover, the fusion between the amino-terminal heterodimerization domain of CBFβ and the C-terminal coiled-coil region of SMMHC creates a novel binding site for AML1, called the AML1 (RUNX1) high-affinity binding domain (HABD) [64]. Therefore, the CBFβ-SMMHC binds AML1 at two sites, resulting in a higher binding affinity for AML1 than wild-type CBFβ [65]. In patients with one allele of the wild-type *CBFB* and one allele of *CBFB*-*MYH11*, AML1 will be preferentially bound to the fusion protein [64,65]. This high-affinity AML1 binding has been proposed to explain the dominant negative role of CBFβ-SMMHC by sequestering AML1 from its targets [64]. However, knock-in mice expressing *Cbfb-MYH11* with a HABD deletion unexpectedly potentiated its leukemogenic activity, developed leukemia faster, even though hematopoietic defects associated with Runx1-inhibition were partially rescued, suggesting that AML1-dominant inhibition may not be a critical step for leukemogenesis by CBFβ-SMMHC [55]. CBFβ-SMMHC was also shown to bind and sequester HIPK2, preventing the critical AML1/p300 phosphorylation to mislocalized CBFβ-SMMHC complexes [66]. Martens and colleagues [67] analyzed inv(16) AMLs by multiple transcriptomic and epigenomic profiles with the aim to investigate whether *CBFB–MYH11* specifically blocks megakaryocyte/erythrocyte differentiation in the context of human hematopoiesis. Findings revealed that CBFβ-SMMHC seems to be involved in transcription deregulation and occupancy replacement of the transcription factors GATA2 (GATA binding protein 2) and KLF1 (Kruppel-like factor 1), interfering with normal differentiation. These results indicate that the attenuating expression of GATA2/KLF1, induced by CBFβ–SMMHC fusion, inhibits primed megakaryopoiesis [67]. On the other hand, a recent paper [68] showed that Gata2 determines its distinct effects in association with *Cbfb-MYH11* in two different stages. Up-regulated *Gata2* gene is important in preleukemic *Cbfb-MYH11* knock-in mice. Heterozygous knockout of *Gata2* in *Cbfb-MYH11* mice delayed leukemia onset. However, despite slower development of leukemia, the *Cbfb-MYH11 Gata2*-deficient mice showed a more aggressive phenotype at the leukemic stage. These findings may reflect the clinical observation of *GATA2* recurrent deletions in relapsed CBFL patients [69]. Therefore, *GATA2* up-regulation contributes to *CBFB-MYH11* leukemogenesis in the early stage, and deficiency could be involved in the relapse/aggressive evolution of CBFL.

## 5. MicroRNA Circuitries Contribute to CBF-Mediated Leukemogenesis

In addition to genetic alterations in the chromatin state that affect gene expression and enrichment for mutations in genes encoding proteins essential for mRNA splicing [70], alterations in the expression of long non-coding RNA (lncRNA) [71], as well as microRNAs (miRNAs), are widely suspected to play a critical role in leukemia initiation and outcome prediction. MicroRNAs are a class of small non-coding RNAs elements (≈20–22 nt) implicated in differentiation of mammalian blood cell lineages through the post-transcriptional modulation of gene expression by binding to the 3′ untranslated region of mRNAs and down-regulation of their translation to protein [72]. The role of lineage-specific miRNAs in hematopoiesis was largely determined from profiling studies that revealed distinct miRNA expression patterns at various stages of hematopoietic development [73,74,75,76]. The expression levels of several miRNA genes show abundant natural variation in nearly all physiological processes involving the formation and maintenance of the human blood hierarchy [77,78]. Human granulocytic differentiation is controlled by *miR-223*, a key member of an integrated regulatory circuit, including *C*/EBPα and NFI-A [23], and *miR-221*/*miR-222* cluster were found to target the oncogene *KIT* [73]. The causal mechanisms and molecular player responsible for the widespread dysregulation of miRNA expression in AMLs, which can function as either oncomiRs or tumor suppressors, are only limitedly known. Specific chromosomal and genetic abnormalities in each AML subtype are likely to contribute to the global non-coding transcriptome. The genomic abnormalities, previously described for protein-coding genes such as chromosomal rearrangements, are found to influence the activity of miRNAs through a variety of mechanisms [27], playing a role in nearly all aspects of AML development [79]. The biological phenomenon where several RNA species regulate one another by competing for binding to a limited pool of shared miRNAs has been proposed as competing endogenous RNAs (ceRNAs) [80]. The potential of the aberrantly overexpressed RUNX1T1 3′UTR, acting as ceRNAs and contributing to t(8;21) alterations of transcriptional balance during AML development, has been addressed by a recent report [81]. Results showed a total of 605 RUNX1T1 ceRNAs significantly enriched in gene ontology (GO) categories mainly associated with leukemia, suggesting the hypothesis that RUNX1T1 may also act as a miRNA sponge in t(8;21) AML, and contributing to explain the complex pattern of gene expression alterations observed in CBFL.

### 5.1. Down-Regulation of miR-222/221 in AML with Deranged Core Binding Factor

One of the most common mechanisms through which miRNA expression dysregulates in AML is yielded by altered transcription factors or oncogenic fusion proteins through epigenetic alterations. Remarkably, the expression of AML1-ETO triggers heterochromatic silencing of genomic regions generating the *miR-223* by recruiting chromatin remodeling enzymes at a RUNX1-binding site on the *pre-miR-223* gene [82]. The AML1-ETO-associated complex resets the *miR-223* gene to a repressed state by changes in chromatin conformation, contributing to the granulocytic differentiation block of the myeloid precursors. Of interest that either ectopic *miR-223* expression, down-regulation of AML1-ETO protein levels, or the use of demethylating agents reactivates *miR-223* expression and restore myeloid differentiation in t(8;21)-AML blasts [82]. Furthermore, the expression level of both KIT mRNA and proteins is much higher in the CBFL, with either wild-type or mutant *KIT*, than in leukemia cells negative for CBF rearrangements [83,84]. Gain-of-function *KIT* mutations, resulting in constitutive tyrosine kinase activity, are significantly enriched in patients with core binding factor leukemia [85,86], and these mutations are associated in t(8;21)-related leukemia with unfavorable outcome [87,88]. Aberrant activation of KIT results in MYC (MYC Proto-Oncogene, BHLH Transcription Factor)-dependent *miR-29b* down-regulation and an increase in Sp1 expression that results in *KIT* overexpression by NFkB transactivation, implicating deregulation of the protein–miRNA network Sp1/NF**k**B/HDAC/miR-29b in KIT-driven leukemia [89]. However, the molecular mechanisms explaining the peculiar association between rearrangements involving the CBF subunits and overexpression of either wild-type or mutant KIT receptor appear much more complex. We reported that CBFL blasts with either t(8;21) or inv(16) rearrangements, characterized by higher expression levels of KIT, display a significantly lower expression levels of *miR-222/221* cluster, a negative modulator of KIT, than non-CBFL blasts. Consistently, the t(8;21)-derived fusion protein induces transcriptional repression of the *pre-miR-222/221* promoter by binding at the evolutionarily conserved RUNX1-binding sites and thus leading to *KIT* overexpression [84].

### 5.2. Epigenetic Silencing of miR-193a Contributes to t(8;21)-Mediated AML

**Figure 5:**
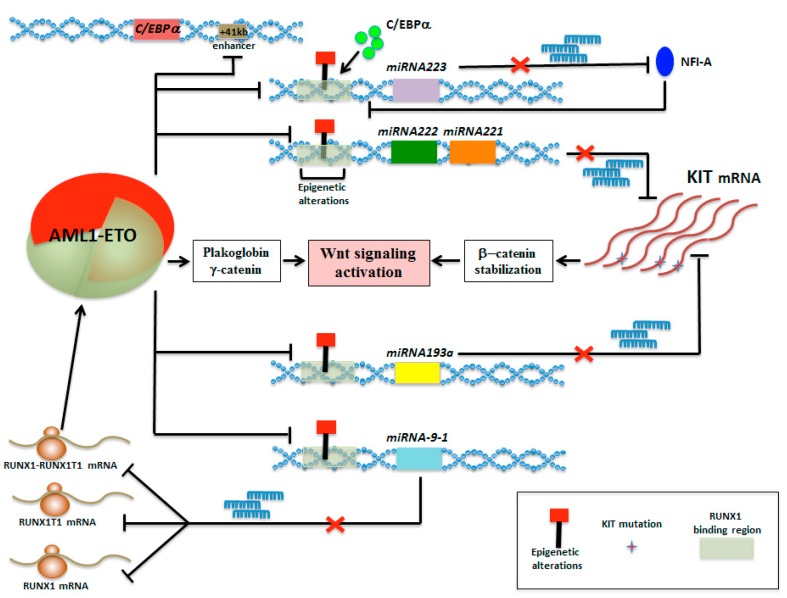
Schematic representation of the AML1-ETO integrated regulatory network comprised micro-RNA minicircuitries promoting Wnt signaling in t(8;21)-AML. AML1-ETO triggers heterochromatic silencing of genomic regions generating the *miR-223, miR-222/221* cluster, *miR-193a*, and *miR-9-1* by recruiting chromatin remodeling enzymes at their specific RUNX1-binding sites; in turn, these miRNA repressions generate differentiation impairments and *KIT* up-modulation that converges with AML1-ETO to activate the Wnt signaling.

Interestingly, *miR-193a* represses the expression of multiple target genes, such as *RUNX1-RUNX1T1*, *DNMT3A* (DNA methyltransferase 3 alpha), *HDAC3* (histone deacetylase3), *KIT*, *CCND1* (B-cell leukemia/lymphoma 1, BCL1), and *MDM2* (transformed mouse 3T3 cell double minute 2) directly, and increases *PTEN* (phosphatase and tensin homolog deleted on chromosome ten) indirectly. AML1-ETO triggers the heterochromatic silencing at the RUNX1-binding sites of *miR-193a* by recruiting chromatin remodeling enzymes and expanding the oncogenic activity of the fusion protein [90]. These further studies add new insights into understanding the concomitant occurrence of CBF genetic rearrangements and overexpression of wild-type or mutant KIT in CBFL, explaining how CBF fusion proteins may therefore maintain expression of several genes (i.e., KIT, WT1) by repressing the expression patterns of their specific miRNAs (Figure 5) [49,84,90]. Another interesting notion is that KIT-ITD mutant cooperates with canonical Wnt signaling pathway, inactivating GSK3β by hyperphosphorylation, which results in increased β-catenin stability [91]. Hence, activation of Wnt signaling plays an important role in KIT-mediated transformation of myeloid cells. Of note, a step forward suggests that activated Wnt/β-catenin signaling induces *RUNX1* transcription mainly through direct β-catenin binding to TBE Site-II, which is located in a highly conserved region within the P1-distal promoter of *RUNX1* [92]. Therefore, Wnt/β-catenin signaling rapidly enhances *RUNX1* expression in leukemia-derived cell lines and human CD34+ hematopoietic cells, suggesting that transcriptional deregulation of translocation-prone genes occurs prior to translocation [93]. Furthermore, recent results highlight the role of Wnt signaling in AML, describing a new rearrangement leading to *WNT10B* overexpression, with the exception of clinical contexts with recurrent cytogenetic abnormalities [94], suggesting a different mechanism promoting Wnt signaling activation in CBFL. In line with these findings, it appears interesting the functional relevance of Wnt signaling induced by CBFL fusion proteins via plakoglobin (γ-catenin) induction [95], which provides a possible explanation of the different molecular circuitry involved in Wnt signaling activation as a common feature of several balanced translocations in AML (Figure 5).

### 5.3. Epigenetic Mini-Circuit AML1-ETO/miR-9-1/miR-383 Contributes to t(8;21) Leukemogenesis

Additionally, new information indicates a feedback minicircuitry through which the expression of *miR-9-1* was decreased by AML1-ETO repression activity, leading to increasing level of RUNX1, RUNX1T1, and RUNX1–RUNX1T1, which are all targeted by *miR-9-1* (Figure 5) [26]. As thanatos-associated protein 10 (THAP10) is a nuclear protein that inhibits myeloid proliferation and promotes differentiation both in vitro and in vivo, AML1-ETO inhibits expression of the tumor suppressor THAP10 directly via epigenetic suppression of the *THAP10* promoter and indirectly through transcriptional activation of *miR-383* in t(8;21) AML, unveiling a novel epigenetic mini-circuitry of AML1-ETO/THAP10/*miR-383* [28]. Many complexities of miRNA biology have already shed additional light on our understanding of how miRNAs function in AML, substantially improving our understanding of how miRNAs synergize within CBFL cells.

## 6. The Genomic Landscape of Core-Binding Factor Acute Myeloid Leukemias

Current treatment guidelines for CBFL with t(8;21) do not take into account heterogeneity in these patients, and thus, all CBFL patients generally receive the same induction and consolidation treatments. Many comprehensive genetic analyses recognize that combination of several genetic alterations is associated with the development of CBFL, and is necessary for a better risk stratification in this leukemia. Although the spectrum of mutations for both CBFL subtypes is similar to the reported signature for AML [96], gene expression and mutation profiling of CBFL identified t(8;21) and inv(16) patients as two distinct subgroups [97], reflecting alternative signals activated in each subtype of CBFL [98]. Moreover, 35% of CBFL patients have two or more mutations in tyrosine kinase (TK) genes coding for pathway effectors (especially *KIT*, *FLT3*, and *RAS* genes); these findings highlight the multiclonality of CBFL. *NRAS* is the most frequently mutated gene in CBFL, more common in *CBFB–MYH11* with a different spectrum of mutations, yet its mutations are not associated with outcome. *KIT* mutations are found in ~40% of CBFL with t(8;21) and ~33% with inv(16); additionally, an enrichment of exon 17 *KIT* mutations has been documented in *RUNX1–RUNX1T1* patients, and are associated with worse outcome [87,99,100,101]. Recent large study created an “International CBF group index for t(8;21)” and validated a new risk scoring system, showing that older age, higher WBC index at diagnosis [102], and KIT D816V/Y mutations were risk factors associated with treatment failure (relapse or death) [103]. These studies strongly support the adverse effect of a *KIT* mutation in the context of CBFL. In addition, a novel finding indicates that pseudodiploidy was also a risk factor in t(8;21). High-risk score patients may benefit from more intensive approaches in the first complete remission (CR1) [103]. Mutations affecting *FLT3*–ITD are present in only 3% of inv(16) AML, whereas they occur in 10% of t(8;21) leukemia patients. In addition to mutations in genes involving TK signaling, alterations in *MGA* (MAX dimerization protein), a negative regulator of MYC signaling, were also recurrently identified in CBFL [104]. Recent results identified CCND2 (cyclin D2) expression as key transmitter of *RUNX–RUNX1T1*-driven AML, promoting cell cycle progression with the cooperation of the transcription factor Activator protein 1 (AP-1), and suggesting new potentially targetable complexes in CBFL [14,105]. Loss-of-function mutations in genes that regulate chromatin-modifying genes (*ASXL1/2*, *EZH2*, *KDM6A*, *BCOR/BCORL1*, *EED*, *SETD2*, *KMT2D*, *KMT2C,* and *CREBBP*) or in genes implicated in the cohesin complex (*RAD21*, *SMC1A*, *SMC3*, *STAG2*) were observed almost exclusively in *RUNX1–RUNX1T1* AML. Cohesin mutations led to a state of increased chromatin accessibility of binding sites for master hematopoietic transcription factors such as AML1 [106]. These findings suggest links between cohesin-mediated alterations in chromatin structure, or chromatin modifiers mutations, and cooperativity with the AML1–ETO fusion oncoprotein, even if cohesin mutations concerned less than 10% of CBFL [15]. CBFL patients with mutations in the above members of the complex, responsible for sister chromatid cohesion during mitosis and DNA repair, lack evidence of aneuploidy or an increase rate of genetic instability without any effect on the outcome. Recently, mutations in *ASXL1* (additional sex combs like 1), *ASXL2* (additional sex combs like 2), *ZBTB7A* (zinc finger and BTB domain conteining 7A), *CCND2*, and *DHX15* (DEAH-box helicase 15) have been frequently identified in *RUNX1–RUNX1T1* but not in *CBFB–MYH11* AML patients [14,107]. *ASXL1* or *ASXL2* truncating mutations, which inhibit myeloid differentiation and induce a myelodysplastic syndrome-like disease in mice [108,109], have been described in ~35% of t(8;21) while are absent in inv(16) AML [15,110,111]. Of interest, chromatin modifier *ASXL1,* as well as cohesin gene mutations, are co-occurring alterations significantly enriched in patients with mutated *RUNX1* AML [112,113]. The nature of cooperating mutations associated with t(8;21)-mediated leukemogenesis is evidenced by additional cytogenetic abnormalities such as trisomy 8 and 4, chromosome 9 deletion, and loss of one of the sex chromosomes [114,115,116]. Increased dosage of the mutated *KIT* (mapped at 4q12) can occur due to trisomy 4, leading to duplication of the mutant *KIT* allele, and suggesting an additional contribution to leukemogenesis [86]. These observations are supported by a higher dosage of N822K *KIT* mutated allele linked to an increased segregation of minichromosomes derived from chromosome 4 that preserve the pericentromeric region containing the *KIT* gene in the t(8;21) positive Kasumi-1 cell line [117]. The most common additional cytogenetic features associated with t(8;21) include loss of either the X or Y chromosome in a disproportionally large number of cases (50–60%), and del(9)(q22) in 15–25% of patients. It has been proposed that haploinsufficiency must be occurring at genes located within shared sequences in the pseudoautosomal regions (PARs) on the X and Y chromosome. A critical event potentially explaining the high incidence of loss of sex chromosomes in t(8;21) may be the loss of *CSF2RA* (colony stimulating factor 2 receptor alpha subunit) gene, encoding for the α subunit of the heterodimeric receptor CSF2 (colony-stimulating factor 2), which control granulopoiesis [118]. However, given that the whole sex chromosome is typically missing and not only the individual *CSF2RA* locus, it is likely that additional haploinsufficient factors on the sex chromosome are acting to enhance *RUNX1–RUNX1T1*-associated leukemogenesis [119]. Sex chromosome loss was reported as a favorable marker for two-year event-free survival (66.9% vs. 43.0%), and in another study showed a modestly favorable, but not significant, effect on disease-free survival (DFS) [103]. Moreover, this last study found that patients with pseudodiploid karyotypes had worse outcome compared with those with hypodiploidy or hyperdiploidy [103]. In contrast, loss of the Y chromosome showed shorter disease-free survival (DFS) for male patients [120].

## 7. Mouse Models for Core Binding Factor Leukemia

The homozygous disruption of *Runx1* or *Cbfb* in murine knockout models exhibits a similar range of abnormalities associated with developmental defects. These common phenotypes include severe hematopoietic defects such as lack of HSCs and progenitors leading to midgestation embryonic lethality between embryonic days (E) 12.5–13.5. Thus, creating mice deficient in *Runx1/Cbfb*, lacking the ability to contribute to definitive hematopoiesis, several research groups have shown that the AML1/CBFβ transcription factor complex is essential in the hematopoietic fate process from the hemogenic endothelial cells. [29,121,122,123]. Moreover, all heterozygous *Runx1-Runx1t1* knock-in mice die around 12.5 days of embryogenesis and fail to establish definitive hematopoiesis [124,125]. This similarity in phenotypes suggests that AML1-ETO effectively neutralizes the normal biologic activity of the AML1/CBFβ transcriptional factor complex and dominantly blocks AML1 activity during early development. Both mouse and in-vitro models have shown that the expression of *Runx1-Runx1t1* or *Cbfb-Myh11* contribute to leukemogenesis but require additional “hits”. An interesting observation that emerged from Cre recombinase-mediated conditional AML1-ETO expression transgenic mice is that the non-leukemic AML1–ETO expressing cells were cytokine-dependent, strongly suggesting that one signaling pathway that may collaborate with AML1–ETO is cytokine-mediated proliferation or survival [13,126]. The first step towards CBFL would consist of the acquisition of genetic alterations, like the CBFL translocations, in modulators of differentiation (class 2 mutations) by the preleukemic clone; the second step would be the acquisition of activating mutations in cell cycle and proliferation controllers (class 1 mutations) such as *KIT*, *FLT3,* or *N-RAS* tyrosine kinases [14,86,127,128,129,130,131], and their cooperation with *Runx1-Runx1t1* or *Cbfb-Myh11* is thought to be crucial for leukemogenesis [132]. CBFβ-SMMHC dominantly represses AML1 function, generates defects in definitive hematopoiesis [133], and predisposes mice to leukemia with cooperating gene mutations [12,134]. Therefore, the fusion protein alone is necessary but not sufficient to cause leukemia, and activating mutations in genes encoding for receptor tyrosine kinases (RTKs) or small GTPase represent the most common genetic events cooperating with CBFL-associated gene fusions [12,126,134]. Although *RUNX1-RUNX1T1* and *CBFB-MYH11* share a common molecular alteration involving the CBF transcription complex, they up-regulate specific signaling pathways essential for stem cell self-renewal and have remarkably different spectra of cooperating mutations [14,95,96,135,136].

The HSCs from adult chimeric mice generated with Cbfb^Cbfb–MYH11/+^ ES cells were found to give rise efficiently to mature erythrocytes, but were unable to differentiate along myeloid and lymphoid lineages [58], suggesting that the differentiation impairment involves the lympho-myeloid primed progenitor (LMPP) as from the revised hematopoietic tree [137]. Knockdown of *Runx1* inhibits the growth and survival of *Runx1-Runx1t1* leukemia cells [138,139]. Moreover, a variant of the AML1-ETO fusion protein, *Runx1-Runx1t19a* (ETO9a), which includes an extra exon 9a of the *Runx1t1* gene that contains C-terminal truncation, was found to be a much more potent inducer of leukemia than the full-length *Runx1-Runx1t1* in mouse retroviral transduction–transplantation model [140]. It has been hypothesized that the deleted region inhibits the leukemogenic potential of AML1-ETO [141]. Interestingly *Runx1-Runx1t1tr*, a C-terminally truncated protein similar to *Runx1-Runx1t19a*, lost the ability to inhibit cell cycle progression of myeloid cells, which may contribute to its enhanced leukemogenic potential [142]. Moreover, the ability to regulate the expression of the *CD44* gene of both RUNX1-RUNX1T1 and its splice variant RUNX-RUNX1T19a links the t(8;21) translocation to the regulation of a cell adhesion molecule involved in the growth and maintenance of the AML blast/stem cells [141]. Novel integrative data analyses, together with siRNA-mediated depletion of AML1-ETO, strongly support the notion that AML1-ETO binds genes associated with the cell structure and cell-cycle progression affecting transcriptional programs associated with myeloid differentiation, proliferation, and self-renewal, in addition to those promoting DNA synthesis [49]. H3K9Ac at AML1-ETO-binding sites show a significant increase after knockdown, which would be compatible with a repressive role of the fusion protein at these sites.

Critical genes such as *CSF2RA* (GM-CSF receptor alpha) and *IL3RA* (IL3 receptor alpha) on human sex chromosomes are localized to syntenic regions on murine 19 chromosome. The receptors for granulocyte-macrophage colony-stimulating factor (GM-CSF), interleukin (IL)-3, and IL-5 all share a common β chain (β_C_) but have a ligand-specific α chain [143]. Matsuura and colleagues [118], in order to explain the selective advantage for sex chromosome loss in t(8;21)-AML, used a transduction/transplantation assay where *Runx1-Runx1t1* was expressed from a retroviral vector in βc-knockout bone marrow cells, which were then transplanted into irradiated wild-type recipient animals. Interestingly, loss of β_C_ in association with AML1-ETO significantly accelerated progression to AML. GM-CSF signaling deficiency is favorable for leukemia development driven by *Runx1-Runx1t1* in mouse models. Moreover, GM-CSF signaling inhibits *RUNX1-RUNX1T1*-mediated leukemogenesis by reducing the self-renewal potential of hematopoietic stem and progenitor cells in murine bone marrow and in human t(8;21) Kasumi-1 cells. These findings suggest an unexpected tumor-suppressor role of GM-CSF in t(8;21) leukemias.

## 8. Molecular Targeted Therapy of CBFL: The Progress and Future Prospect

Individual genomic characterization may be useful to suggest or not targeted interventions, such as the use of RTK inhibitors, for instance dasatinib [144,145] or midostaurin [146], in patients carrying activating *KIT* and/or *FLT3* gene mutations. *KIT* mutations are common in CBFL and have been associated with worse prognosis (shorter disease-free survival, relapse-free survival, event-free survival, overall survival) [147]. A phase III study of gemtuzumab ozogamicin (Mylotarg®; Pfizer/Wyeth-Ayerst Laboratories) showed that GO abrogated the negative prognostic effect of exon 17 (E17) mutations in treated patients [148]. Moreover, the outcome of patients harboring *KIT* mutations in the Cancer and Leukemia Group B (CALGB) study, appeared not to be worse than that of patients with *KIT* wild-type after treatment with dasatinib, implying that a potentially adverse impact of *KIT* mutations might be abrogated in treated patients [144]. Conversely, within the German-Austrian AML Study Group trial (AMLSG 11- 08 trial), no favorable impact after dasatinib administration was noted for patients with concurrent *KIT* mutation [145]. Although validation by other studies is needed, the rationale to combine dasatinib with other compounds was supported by data from a mouse model of t(8;21)-positive and KIT-mutated leukemia, where the combination of dasatinib with cytarabine prolonged the survival of the animals compared to the exposure with these drugs as single agents [146,149]. As acquisition of spliceosome gene mutations result to be the initiating events in clonal hematopoiesis, the use of spliceosomal modulation to induce synthetic lethality in splicing factor mutant hematological malignancies, currently being tested in an early phase clinical trial, is quite exciting [150]. CBFL is a still heterogeneous disease entity, and for better characterization of CBFL risk heterogeneity in the spirit of precision medicine, it is necessary to elucidate the combinations of genomic abnormalities and clonal evolutions using refined high-resolution genomic analysis to develop new treatment strategies for CBFL.

## 9. Conclusions

Genetic definition of CBFL patients using deep sequencing approaches illustrate that we are only beginning to understand how fusion proteins involving the CBF are integrated into the molecular networks of transcriptional and epigenetic regulators. Despite the generation of in vivo models helped us to understand how CBFL originates and propagates, we can imagine a future where the understanding of how expression and CBF fusion proteins activity are modulated during myeloid leukemia transformation and progression will trigger a true progress to translate into the clinic. Such achievements will become extremely useful, in a view of individual treatment on the basis of defined targets. Future studies will be required to identify which CBFL patients could benefit from therapy by each molecular drug combination targeting a specific pathway.
